# The African Female Breast Cancer Epidemiology Study Protocol

**DOI:** 10.3389/fonc.2022.856182

**Published:** 2022-04-13

**Authors:** Emmanuel R. Ezeome, King-David T. Yawe, Omobolaji Ayandipo, Olawale Badejo, Sally N. Adebamowo, Benerdin Achusi, Adeola Fowotade, Gabriel Ogun, AFBRECANE Research Group, Clement A. Adebamowo

**Affiliations:** ^1^Department of Surgery, College of Medicine, University of Nigeria, Enugu, Nigeria; ^2^Oncology Center, University of Nigeria Teaching Hospital, Enugu, Nigeria; ^3^Department of Surgery, University of Abuja Teaching Hospital, Abuja, Nigeria; ^4^Department of Surgery, University College Hospital, Ibadan, Nigeria; ^5^Department of Pathology, National Hospital, Abuja, Nigeria; ^6^Department of Epidemiology and Public Health, and Greenebaum Comprehensive Cancer Center, University of Maryland School of Medicine, Baltimore, MD, United States; ^7^Department of Anatomic Pathology, Federal Medical Center, Abuja, Nigeria; ^8^Department of Medical Microbiology, University College Hospital, Ibadan, Nigeria; ^9^Department of Pathology, University College Hospital, Ibadan, Nigeria; ^10^Institute of Human Virology Nigeria, Abuja, Nigeria

**Keywords:** breast cancer, female, Africa, genomics, epidemiology

## Abstract

Breast cancer is now the commonest cancer in most sub-Saharan African countries. Few studies of the epidemiology and genomics of breast cancer and its molecular subtypes in these countries have been done. The African Female Breast Cancer Epidemiology (AFBRECANE) study, a part of the Human Heredity and Health in Africa (H3Africa) initiative, is designed to study the genomics and epidemiology of breast cancer and its molecular subtypes in Nigerian women. We link recruitment of breast cancer cases at study sites with population-based cancer registries activities to enable ascertainment of the incidence of breast cancer and its molecular subtypes. We use centralized laboratory processing to characterize the histopathological and molecular diagnosis of breast cancer and its subtypes using multiple technologies. By combining genome-wide association study (GWAS) data from this study with that generated from 12,000 women participating in our prospective cohort study of cervical cancer, we conduct GWAS of breast cancer in an entirely indigenous African population. We test associations between dietary intakes and breast cancer and focus on vitamin D which we measure using dietary intakes, serum vitamin D, and Mendelian randomization. This paper describes the AFBRECANE project, its design, objectives and anticipated contributions to knowledge and understanding of breast cancer.

## Introduction

While the incidence of breast cancer has stabilized in high-income countries (HIC), it is rising in low income countries (LIC) that hitherto had low incidence (1). The WHO and the International agency for Research on Cancer (IARC) estimate that by the year 2040, there will be 3.06 million new cases of breast cancer every year and most of them will be in LIC (2). This is due to the epidemiological transition going on in these countries and the increase in the prevalence of known risk factors such as obesity, hormone replacement therapy (HRT), as well as increasing use of early detection and screening methods (3–7). Nevertheless, these factors do not account for all the increase in breast cancer incidence, while the specific contributions of some of them such as mammographic screening, hormone replacement treatments, and hormonal agents are hotly debated (5, 8–16).

In contrast to developed countries, there has been few systematic studies of breast cancer in sub-Saharan Africa. Studying the epidemiology and genomics of breast in developing countries like Nigeria will enable identification of critical environmental and genetic risk factors and improve our knowledge of the causes of its rising incidence. The AFBRECANE study is funded by the Office of the Director of the US National Institutes of Health (NIH) and the National Human Genome Research Institute (NHGRI) under the Human Heredity and Health in Africa (H3Africa) initiative for five years starting in November 2018. The project is designed to study the genetic and environmental risk factors of breast cancer and its subtypes in an indigenous African population.

The primary objectives of the AFBRECANE study are to determine the incidence and prevalence of total and molecular subtypes of breast cancer in Nigerian women and to characterize their secular trend in Nigeria. We achieve this through combination of case-control and cancer registration research methods through enrollment at our study sites and linkage with the Population Based Cancer Registries (PBCR) in the cities where participants are being enrolled in Nigeria. In this study, we link epidemiological risk factors’ data, cancer registry databases, clinical information and biorepository of tissues for breast cancer research in Nigeria. We engage multiple methods for determination of molecular subtypes of breast cancer in tissue biopsies and fine needle aspirates and evaluate determinants of discordance between the different methods.

Another objective of the AFBRECANE project is to conduct GWAS of breast cancer and its molecular subtypes to look for mutations associated with breast cancer and its subtypes in indigenous African populations. In order to efficiently achieve this objective, the project takes advantage of the GWAS data from H3Africa funded African Collaborative Center for Microbiome and Genomics Research (ACCME) project which enrolled women who volunteered for cervical cancer screening and were free of cancer at baseline. The use of an enriched microarray chip designed by the H3Africa Consortium for this GWAS study promises to increase the likelihood of detecting new variants that are significantly associated with breast cancer in this African population. We will use Mendelian Randomization to test associations between SNPs associated with serum vitamin D and breast cancer in Nigerian women.

The third objective of the AFBRECANE project is to evaluate associations between Nigerian diets and their food and nutrients composition, and breast cancer. Using our validated Food frequency questionnaire (FFQ), Food composition database (FCD), and Food picture book (FPB), we characterize the dietary intakes of our participants. Further, we specifically examine dietary and serum levels of vitamin of D, as well as use Mendelian Randomization (MR) of SNPs associated with serum levels of vitamin D, and risk of breast cancer in this population in Africa.

## Methods And Analysis

### Study Design and Rationale

In the AFBRECANE project, we establish clinical research sites in 3 cities in Nigeria and link these sites with the population-based cancer registries (PBCR) covering the population in these cities. This enables us to use cancer registration methods to ascertain the incidence of breast cancer and its molecular subtypes in this population. Further by enrolling population based controls, we evaluate the role of demographics, lifestyle, and diet in the epidemiology of incident breast cancer and its subtypes in Nigerian women (17–19).

#### Molecular Subtypes of Breast Cancer in Africa

Several studies have reported that the growth in incidence of breast cancer in developed countries was largely due to increases in incidence of Luminal types A and B (hormone receptors rich) breast cancers, while the incidence of other subtypes including triple negative breast cancer (TNBC) remained stable (8, 20). In a study using the SEER database, the proportion of Estrogen receptor (ER) positive and Progesterone receptor (PR) positive breast cancers among 40 to 69 years old women increased from 75.4% to 77.5% (p-value = 0.0002) and from 65.0% to 67.7% (p-value < 0.001) between 1992 and1998 (20). In contrast, several authors have suggested that the recent increase in breast cancer incidence in Africa is mostly due to rising prevalence of hormone-receptor poor tumors (21, 22). To date it has been difficult to test this hypothesis because of lack of population-based cancer registry (PBCR) and immunohistochemistry laboratories in most African countries (19). The AFBRECANE study resolves these challenges by working with the Nigerian National System of Cancer Registries and using a centralized laboratory for specimen analyses to evaluate secular differences in incidence of molecular subtypes of breast cancer (17–19).

#### Dietary Intakes and Breast Cancer Risk in Africa

The growing adoption of western lifestyles including dietary patterns has been touted as one of the risk factors for the rising incidence of breast cancer in African populations (16, 23–26). To date, there has been no robust study of the associations between dietary intakes and breast cancer in indigenous African populations (27). Very few of the studies done so far used validated Food Frequency Questionnaires (FFQ), the paradigmatic tool for nutrition epidemiology of complex diseases (28, 29). Nutrition epidemiology research also requires methods for obtaining information on portion sizes because of poor standardization of serving sizes in Africa, and food composition databases (FCD) of African foods to ensure correct computation of nutrient values. None of the studies of diets and breast cancer done in Africa till date has found strong associations between any African diet, their food and nutrient compositions, and the risk of breast cancer. In the AFBRECANE study, we use validated FFQ, FCD of Nigerian foods and a Food Picture Book (FPB) to obtain information on food intakes for studies of their association with breast cancer.

#### Vitamin D and Breast Cancer Risk

Studies from developed high-income countries have suggested that there are strong associations between dietary intake of vitamin D or serum levels of vitamin D and breast cancer risk and outcomes (30–34). This association may be stronger in premenopausal women, patients with advanced breast cancer and in triple negative breast cancer (35). Further, vitamin D status may explain some of the racial disparities in incidence and outcomes of breast cancer (36–40). African-Americans with low mean serum vitamin D levels had higher risk of breast cancer, compared to European Americans. Most Sub-Saharan Africans have low overall intake of vitamin D and recent studies have shown high prevalence of hypovitaminosis D in Africa populations, despite high levels of sun exposure (41). Skin tones, which may influence bioavailable vitamin D levels of Africans, also vary significantly and there has been no previous study of vitamin D and breast cancer that adjusted for skin tones. There has been no previous study of vitamin D and its association with breast cancer risk in indigenous Africans. The AFBRECANE study will fill this gap.

In addition to evaluating associations between vitamin D levels derived from nutrient levels in dietary intakes and serum vitamin D measurements, we also evaluate the association between vitamin D and breast cancer risk by Mendelian Randomization using a combination of SNPs as instrumental variables (IV). This overcomes the problems of reverse causation and confounders in assessing any causal relationships between vitamin D and breast cancer risk (42–51). 1,25-dihydroxyvitamin D (1,25(OH)2D), the natural ligand of the vitamin D receptor (VDR), is a transcription factor that is expressed in more than thirty-five tissues. More than 200 genes containing VDR-response elements and their expression is affected by 1,25(OH)2D. Mutations in one or more of these genes may affect the biological activity of vitamin D and its effect on disease occurrence. SNPs that are known to be associated with serum 25-hydroxyvitamin D levels would be used as instrumental variables to estimate the effect of vitamin D on breast cancer risk. Since SNP genotypes are determined at birth, they are not influenced by potentially confounding variables, and so the effect estimate from multiple regression analysis should be unaffected by reverse causation.

#### Genome-Wide Association Study (GWAS) of Breast Cancer in Indigenous Africans

Previous studies have described associations between some SNPs and breast cancer in African populations. Some of these SNPs have been reported in non-African ancestry populations while others appear to be specific to Africans (52). Although there have been several case-control GWAS of breast cancer in African ancestry populations, none has been solely of Africans living in Africa (53–80). In the previous studies, the genotyping array chips used were not designed to account for African populations. Given the genomic heterogeneity of Africans, it is probable that GWAS of breast cancer in indigenous African population using microarray chips enriched for African populations will identify more unique genomic loci associated with breast cancer and its molecular subtypes in African women. The AFBRECANE study provides an opportunity to conduct GWAS of breast cancer and its molecular subtypes in indigenous African population using chips enriched for genetic variants in African populations.

### Participants

**Figure 1:**
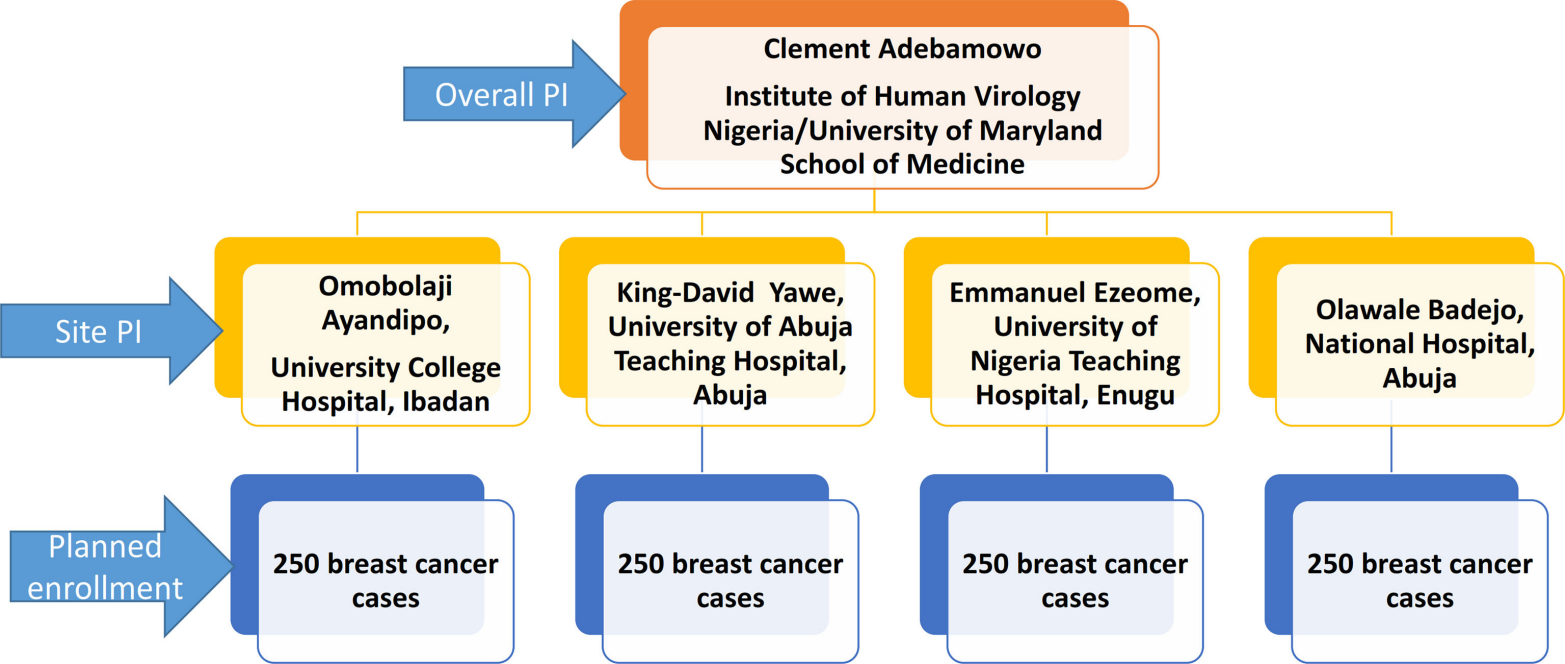
Organogram and Coordination of the AFBRECANE Study.

The cases in this study are women aged 18 years and older with clinical and histological diagnosis of breast cancer. A total of 1000 cases will be enrolled over five years from clinical sites across Nigeria. Enrollment commenced in November 2018 at National Hospital Abuja and University of Abuja Teaching Hospital Gwagwalada in central Nigeria, University College Hospital Ibadan in Western Nigeria and University of Nigeria Teaching Hospital, Enugu in Eastern Nigeria. The sites were selected based on their case volume, representation of different geographical zones of Nigeria as well as availability of population-based cancer registries in those areas. **Figure 1** displays the organogram of the AFBRECANE study and how it is coordinated by the PI of the study. The research associates report cases enrolled to the cancer registrars in these cities to ensure more complete registration of breast cancer cases by the PBCR. Breast cancer patients identified by the cancer registrars are also invited to participate in the study. Breast cancer patients who opt not to participate in the study contribute to registry data and this enables us to compute the breast cancer incidence in the registries catchment areas. After obtaining informed consent from those willing to participate, research associates conduct confidential and structured face-to-face interviews in the language preferred by the participant. Patients who are too sick to participate or refused to give informed consent are excluded from the study.

For the GWAS component of this study, control participants are the ~12,000 women who are enrolled in the ACCME cohort study. These women had no history of any cancer at enrollment, were HIV negative by self-report and had given broad informed consent for future unspecified use genomic research. The profile of participants in the ACCME cohort has been previously described (81). To generate matching controls for epidemiological case-control analyses, we randomly select women in the ACCME cohort who are within +/- 2.5 years’ age of the breast cancer cases enrolled in the AFBRECANE study. Ethical clearance for this study was obtained from the National Health Research Ethics Committee of Nigeria and the Institutional Review Board (IRB) of the University of Maryland School of Medicine, Baltimore. Each patient gave broad informed consent for the research and the use of the data for future research.

Breast cancer cases are followed up at regular clinic appointments after enrollment until end of study or loss to follow-up. Participants data are stored in REDCap database with paper forms for back-up. The contact details of participants and their next of kin are collected and used for following up. Patients are required to inform the next of kin of their participation in this study and obtain consent of the next of kin so they can be contacted if we cannot reach the participant.

Research associates administer the AFBRECANE study questionnaire earlier developed for breast cancer studies in the Nigerian population. Data collected include socio-demographic data, lifestyle data, dietary intakes data, medical history, obstetrics, and gynecology history of participants (**Table 1**). The associates use our validated semi-quantitative FFQ assisted by the FPB to record participants dietary history (82, 83). They measure participants’ height, weight, waist and hip circumferences, and body shape information (somatotype) per standard protocol. All data are entered into REDCap databases (84, 85). Standard cancer registry methods were used to identify all population based diagnosis of incident breast cancer in the catchment areas of the two PBCR as previously described (18, 86, 87). The Nigerian National System of Cancer Registries Data Abstraction form are used to collect cancer registries data and the CanReg5^®^ open source software is used to check the data and analyze the registries data.

**Table 1:** Data collected by questionnaire.

Variable	Baseline	Follow-up
Demographics	+	
Date and place of birth	+	
Individual, parental and grandparents’ ethnicity	+	
Languages spoken by individuals and parents	+	
Religion	+	
Education and occupation	+	
Marital status and arrangement	+	
Current address and location of longest residence/urban vs rural	+	
Socio-economic data	+	
Household income and ownership	+	
Source of drinking water and feces disposal	+	
Source of cooking fuel and separate room for cooking	+	
Household material goods	+	
Physical activity	+	
Time/week spent on activities at home	+	
Time/week spent on activities away from home	+	
Usual walking pace	+	
Number of flights of stairs climbed daily	+	
Number of days spent exercising/week	+	
Alcohol, cigarette smoking and tobacco use	+	
Age when use of each products became regular	+	
Measure of each product use/daily and total duration of use	+	
Preferred type/brand of each product	+	
Start or successfully quit use of each product	+	
Exposure to secondhand smoke	+	
Food frequency questionnaire		
Average dietary intake of food and beverages over the past year		
Reproductive history	+	
Menstrual history including age of start/stop of regular periods	+	
Contraceptive history including type/duration of use	+	
Pregnancy history/pregnancy outcome and child feeding practice	+	
Medical history	+	
Treatment		+
Impact of treatment		+
Use of alternative and complementary treatments		+

The breast cancer cases are staged using the American Joint Committee of Cancer’s classification of Tumor, Node and Metastasis (TNM). All the patients receive clinical examination of the breasts, chest x-rays, abdominal ultrasound, and bone scans. Breast biopsies are done using core biopsy needles, excisional or incisional biopsies as indicated. One breast tumor sample is inserted into PAXgene^®^ Tissue Fixative which ensures rapid penetration and fixation in 2 to 4 hours. The warm ischaemic time is recorded. The sample is inserted into PAXgene^®^ Tissue Stabilizer to stabilize molecular contents and preserve morphology. A second sample are stored in -80°C. All samples were transported to the ACCME Laboratory at the Institute of Human Virology of Nigeria (IHVN), Abuja, Nigeria where two sets of slides are prepared.

Slides are prepared according to standard histology and immunohistochemistry procedures in the ACCME Laboratory, IHVN Abuja, Nigeria and the results are reported by the study pathologist. The estrogen, progesterone, and HER2 status of the tumors were graded using <1% positivity for cut-off as recommended by the American Society of Clinical Oncology (ASCO)/College of American Pathologists (CAP). Scores of 0, 1, 2, 3 are assigned to the tumors based on the intensity of staining. Additional molecular subtyping of the breast tumors is also done using RT-PCR methods to measure four breast somatic biomarkers, *ESR1, PGR1, ERBB2*, and *MKi67* messenger RNAs (mRNAs), and one reference gene (*CYFIP1*) (88). The results of the molecular subtyping tests are compared and only concordant classifications are accepted. Slide images from discordant IHC tests are captured using Grundium Ocus^®^40 microscope scanners and evaluated at University of Maryland School of Medicine. Cases that are persistently discordant will be analyzed separately.

**Figure 2:**
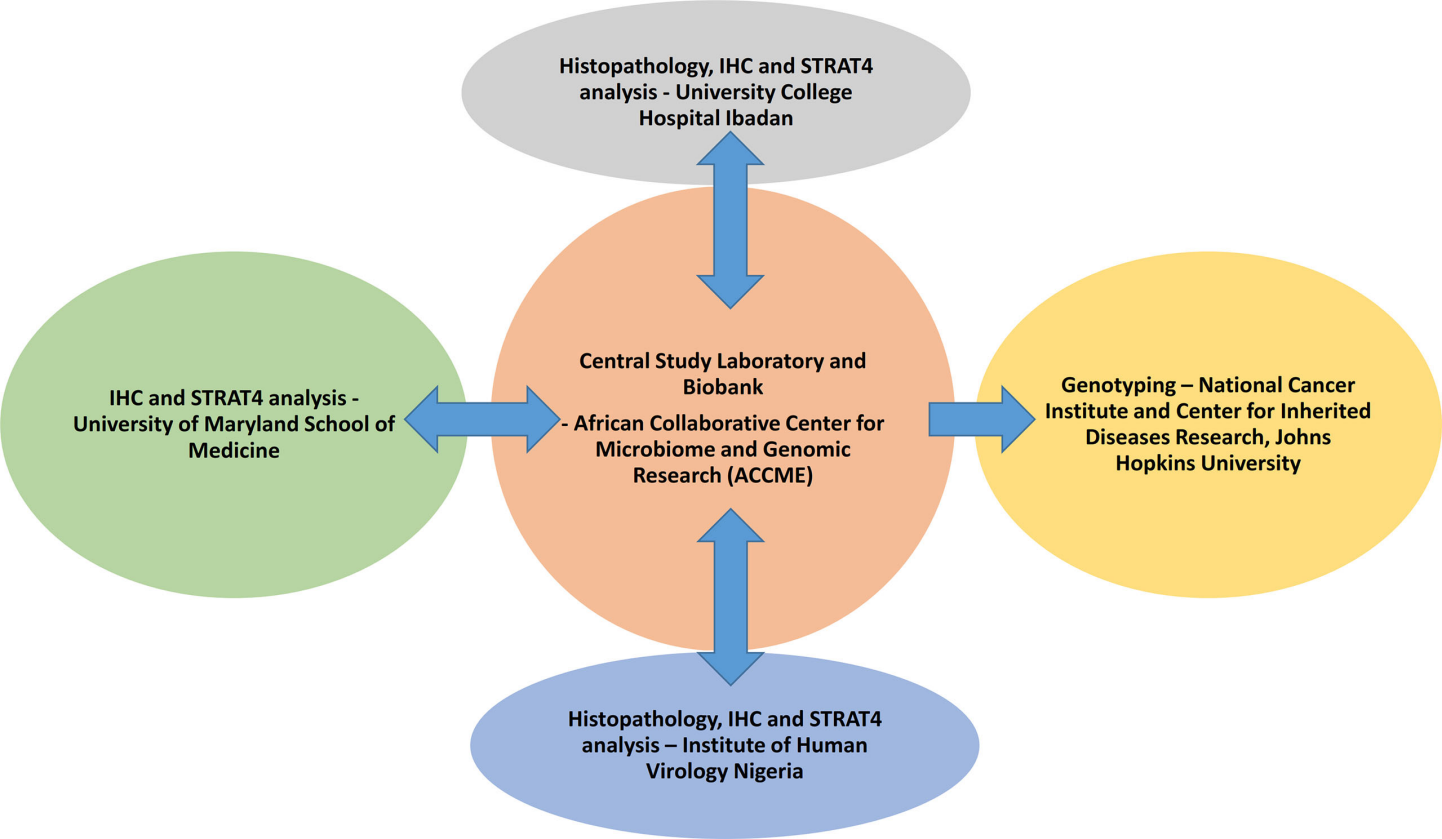
Components of the AFBRECANE study conducted at different sites within and outside Nigeria.

Blood samples are collected from the study participants and separated into plasma, buffy coat, and blot clots which are stored -80°C. Germline DNA were extracted from the buffy coat using Qiagen Qiacube HT^®^ in the ACCME Laboratory in Nigeria. Plasma samples are used for standard chemistry, trace metals, lipids, and biomarkers, and blood clots are used for analysis of fatty acids. **Figure 2** displays the components of the study conducted at different sites within and outside Nigeria.

## Data Analysis And Quality Assurance

All data are entered into Redcap database hosted at the IHVN (84, 85). Research associates interview patients and enter epidemiological and clinical data into tablet computers. To improve quality of laboratory analyses and enhance reproducibility, we ensure that the study is done to a very high standard and implement several quality assurance activities (89). Use of Paxgene^®^ tissue system for collection, handling, transport, and fixation of breast cancer samples reduces pre-analytic variation in tissue handling (90–92). All immunohistochemistry is done in a single central laboratory in Nigeria. We use the same hormone-receptor primary antibodies and clones [Estrogen Receptor Clone 6F11 Ready-To-Use Primary Antibody, Her2/Neu (SP3) Rabbit Monoclonal Antibody, and the Bond™ Ready-to-Use Primary Antibody for Progesterone Receptor (16)] as the Department of Pathology, University of Maryland School of Medicine (93). We keep detailed record of clones and lot numbers of the primary antibodies (94, 95).

Recruitment of patients into the project started in November 2018. As of May 2021, we have enrolled 506 breast cancer cases and in **Table 2**, we present the analysis of their characteristics compared with matching controls from the ACCME cohort. The mean (SD) age of the controls was 47.6 (12.8) years while that of cases was 48.7 (12.0) years. Breast cancer cases had later age of onset of menarche, positive family history of breast cancer in a first degree relative, fewer full-term pregnancies, higher prevalence of nulliparity, history of use of oral contraceptives, were more likely to be pre-menopausal and had similar breast feeding history compared to controls. Breast cancer cases weighed less, were taller, and had lower BMI compared to controls. The molecular subtype patterns of the breast tumors are 51.6% ER positive, 34.4% ER negative, 40.7% PR positive, 43.5% PR negative, 66.8% HER2 positive and 16.2% HER2 negative.

**Table 2:** Baseline characteristics of participants in the AFBRECANE project in Nigeria, 2020.

Breast cancer				
	Control		Invasive case	
Age (years)	47.6	(12.8)	48.7	(12.0)
Highest level of education received				
No formal schooling	79	15.6%	38	7.5%
Koranic school only	6	1.2%	4	0.8%
Vocational only	0	0.0%	2	0.4%
Did not complete primary school	18	3.6%	14	2.8%
Completed primary school only (6 years)	50	9.9%	76	15.0%
Some high school (7-11 years)	31	6.1%	48	9.5%
Completed high school (12 years)	49	9.7%	94	18.6%
Post high school education but not university	90	17.8%	117	23.1%
Completed university education	117	23.2%	84	16.6%
Postgraduate degree	39	7.7%	29	5.7%
Some postgraduate school	26	5.1%	0	0.0%
Age at menarche (years)	14.4	(1.9)	14.6	(2.1)
Family history of breast cancer in a first degree relative				
No	0	0.0%	479	94.7%
Yes	0	0.0%	27	5.3%
Don’t know	506	100.0%	0	0.0%
Number of full-term pregnancies	3.6	(2.4)	3.3	(2.2)
Nulliparous v parous				
nulliparous	64	12.6%	65	12.8%
1+ full term pregnancies	442	87.4%	441	87.2%
Ever breastfed				
No	71	14.0%	71	14.0%
Yes	435	86.0%	435	86.0%
Use of oral contraceptives (OC)				
never	425	84.0%	371	73.3%
ever	79	15.6%	135	26.7%
cannot recall	2	0.4%	0	0.0%
Menopausal status				
pre/peri	274	54.2%	293	57.9%
post (postmenopausal: last menstruation more than 12 months before reference date)	207	40.9%	213	42.1%
don’t know	25	4.9%	0	0.0%
Weight (kg)	77.2	(17.4)	70.9	(15.7)
Adult body height (cm)	159.5	(6.5)	161.2	(7.2)
Body mass index at interview/questionnaire in kg/m2	30.2	(6.3)	27.3	(5.7)
Estrogen receptor status of index tumour				
negative			174	34.4%
positive			261	51.6%
not available			71	14.0%
Progesterone receptor status of index tumour				
negative			220	43.5%
positive			206	40.7%
not available			80	15.8%
Human epidermal growth factor receptor 2 status of index tumour				
negative			338	66.8%
positive			82	16.2%
not available			86	17.0%

## Discussion

### Scope of the Project and Future Directions

The AFBRECANE project will contribute to global knowledge about the epidemiological determinants of incident total and molecular breast cancer types, particularly of variants like triple negative breast cancer that have poor outcomes in indigenous African populations. The project will contribute to knowledge on the associations between indigenous African diets and breast cancer risk. The project focuses in a powerful way on the association between vitamin D and the risk of breast cancer by using different strategies to evaluate vitamin D intakes. Identification of genomic risks of breast cancer in African populations like Nigerians will clarify the roles of genomic variants that have been identified in non-African populations. Other research questions that the AFBRECANE study will address are listed on **Table 3**. The database of tissue samples, clinical details and genomic data collected in this cohort will help future genomic studies, data science studies and epidemiological studies of breast cancer in general and among Africans in particular. Future research will identify the functional relevance of the genes identified in this project and the results may lay the foundation for vitamin D prevention trials in breast cancer.

**Table 3:** Other research questions to be answered by AFBRECANE project.

1	Link epidemiological risk factors, cancer registry, clinical information databases and virtual biorepository of tissues for breast cancer research in Nigeria.
2	Validate modern rapid molecular biology-based tests for determination of molecular subtypes of breast cancer in tissue biopsies and fine needle aspirates.
3	Evaluate determinants of discordance between new molecular biology-based tests of breast cancer immunophenotypes and IHC
4	Compare the pattern of germline epigenetic changes in different stages of breast cancer
5	Evaluate associations between germline Human Endogenous Retrovirus K (HERV-K) clinical, pathological, immunophenotypes and tumor-infiltrating lymphocytes (TIL) in breast cancer biopsies.
6	Breast metabolomics for identification of breast cancer biomarkers for diagnosis, prognosis, prediction and personalized treatment for breast cancer.

Afbrecane Study Group: AFBRECANE Research group is part of the H3Africa Consortium.

### What Are the Main Strengths and Weaknesses?

AFBRECANE project is one of the few large case-controlled study of genomic data on breast cancer from solely indigenous Africa populations. The detailed collection of epidemiological, dietary and nutritional information in the database promises to give insight to the role of environmental and genomic variables in the development of breast cancer and how these have impacted the changing trend in breast cancer incidence both locally and globally. The detailed genomic characterization presents the potential for discovery of new genetic mutations and variants of known mutations that may be active within the African population given the genetic diversity of the African populations.

This is also the only breast cancer study in African populations that includes use of validated FFQ supported by FPB to capture portion size information and a native African FCD. The focus on the role of vitamin D and breast cancer risks includes three independent methods of characterizing vitamin D intakes and adjustment of skin tones which determines the impact of sun exposure on endogenous vitamin D levels. The project is therefore likely to contribute unique insights into understanding any associations between vitamin D and breast cancer in this population.

The main limitation of this cohort is that we plan to enroll 1,000 breast cancer cases, and this sample size may give adequate power for total breast cancer analysis but it may prove insufficient for exploration of some of our hypothesis including GWAS of molecular subtypes of breast cancer. We are enrolling participants from Nigeria only and this may impact the generalizability of our results to other parts of Africa.

### Training and Capacity Development

Built into the AFBRECANE study are capacity development programs in Epidemiology, Data science, Genomics and Breast cancer research. Both pre-doctoral and post-doctoral students are currently working on different aspects of the project, including the data science, the sample analysis, and the epidemiological correlates.

### Collaborations

A key component of the AFBRECANE project is the collaboration with other research projects in Africa and internationally. AFBRECANE project is part of the H3Africa consortium and is contributing data and biospecimen to the H3Africa repositories in accordance with the guidelines. We are also collaborating with the NCI on the Confluence project which aims to conduct GWAS of 300,000 breast cancer cases and 300,000 controls in order to uncover breast cancer genetic risk factors.

We currently collaborate with Cepheid Corporation to validate the STRAT4 GeneXpert test for molecular typing of breast cancer. STRAT4 uses GeneXpert technology to quantitatively classify the molecular subtypes of breast cancer as an alternative to traditional IHC testing. This will be responsive to the challenge of immunohistochemistry testing in Africa and improve access to these results for management and prognostication of breast cancer.

### Can I Get Hold of the Data? Where Can I Find Out More?

The project data release plan will be in accordance with the H3Africa data storage and release policy (96). Access to the data is granted to researchers through the Database Access Committee (DBAC) of H3Afrca and investigators can access the database one year after the end of the project. Genotyping and phenotyping data will be deposited in dbGap (https://www.ncbi.nlm.nih.gov/gap/) More information on the data access protocol can be obtained at the H3Africa data archive @ https://www.h3abionet.org/resources/h3africa-archive. Specific inquiries and proposals for collaboration can be directed to the PI of the project. (cadebamowo@som.umaryland.edu).

## Federal Capital Territory Abuja site group

1. Oge Ikwueme
2. Ayotunde Famooto
3. Tolu Gbolahan
4. Mary Ajayi
5. Mary-Favour Edet
6. Ayobami Ademola
7. Yinka Owoade
8. Faithful Nze
9. Temitayo Oladimeji

## Enugu site Group

10. Robinson Ugwu
11. Mr. Agbalu Ifeanyi Sebastian

## Ibadan site group

12. Temilola Yusuf
13. Tobi Oyediran
14. Adeola Akintola
15. Temitope Olayinka
16. Julius Adediji
17. Chibuzor Nkwodimmah
18. Bisola Famooto
19. Banke Ipadeola

## Consultants

1. Prof. Emmanuel Ezeome
2. Prof. King-David Terna Yawe
3. Dr. Badejo Olawale Ayodele
4. Dr. Abike Fowotade
5. Dr. Omobolaji. O. Ayandipo
6. Dr. Gabriel Ogun
7. Dr. Izuu Achuzi

## Cancer Registries Directors

1. Dr. Festus Igbinoba
2. Dr. Theresa Otu
3. Prof. Emmanuel Ezeome

## Cancer Registrars

1. Chinyere Chukwubuike
2. Henry M. Kumai
3. Ann Okoroafor

## Data Availability Statement

The raw data supporting the conclusions of this article will be made available by the authors, without undue reservation.

## Ethics Statement

The studies involving human participants were reviewed and approved by: 1. Institutional Review Board, University of Maryland School of Medicine, Baltimore; 2. National Health Research Ethics Committee of Nigeria, Federal Ministry of Health, Nigeria. The patients/participants provided their written informed consent to participate in this study.

## Author Contributions

Conceptualization: CA, AFBRECANE Research group. Data curation: EE, K-DY, OA, OB, BA, AF, GO, CA, AFBRECANE Research group. Formal analysis: EE, SA, and CA. Funding acquisition: EE, K-DY, MA, OB, SA, and CA. Methodology: EE, K-DY, OA, OB, SA, BA, AF, GO, CA, AFBRECANE Research group. Project administration: EE, K-DY, MA, AF, and CA. Visualization: EE, K-DY, OA, OB, SA, BA, AF, GO, and CA. Writing–original draft: EE, SA, and CA. All authors contributed to the article and approved the submitted version.

## Funding

The project is supported by the African Female Breast Cancer Epidemiology (AFBRECANE, U01HG009784) and the African Collaborative Center for Microbiome and Genomics Research (ACCME U54HG006947) grants from the National Institute of Health Office of the Director/National Human Genome Research Institute. Additional support was received from the Maryland Department of Health’s Cigarette Restitution Fund, the University of Maryland School of Medicine Greenebaum Comprehensive Cancer Center Support Grant (National Cancer Institute Award Number: P30CA134274), and the American Cancer Society Institutional Research Grant (IRG-18-160-16).

## Conflict of Interest

The authors declare that the research was conducted in the absence of any commercial or financial relationships that could be construed as a potential conflict of interest.

## Publisher’s Note

All claims expressed in this article are solely those of the authors and do not necessarily represent those of their affiliated organizations, or those of the publisher, the editors and the reviewers. Any product that may be evaluated in this article, or claim that may be made by its manufacturer, is not guaranteed or endorsed by the publisher.
